# PBF-LB/M of Low-Alloyed Steels: Bainite-like Microstructures despite High Cooling Rates

**DOI:** 10.3390/ma15176171

**Published:** 2022-09-05

**Authors:** Dominic Bartels, Tobias Novotny, Andreas Mohr, Frank van Soest, Oliver Hentschel, Carsten Merklein, Michael Schmidt

**Affiliations:** 1 Institute of Photonic Technologies (LPT), Friedrich-Alexander-Universität Erlangen-Nürnberg (FAU), Konrad-Zuse-Straße 3/5, 91052 Erlangen, Germany; 2 Collaborative Research Center CRC 814—Additive Manufacturing, 91052 Erlangen, Germany; 3 Erlangen Graduate School in Advanced Optical Technologies (SAOT), Friedrich-Alexander-Universität Erlangen-Nürnberg (FAU), Paul-Gordan-Straße 6, 91052 Erlangen, Germany; 4 Schaeffler Technologies AG & Co. KG, Industriestraße 1-3, 91074 Herzogenaurach, Germany; 5 Deutsche Edelstahlwerke Specialty Steel GmbH & Co. KG, Oberschlesienstraße 16, 47807 Krefeld, Germany

**Keywords:** PBF-LB/M, selective laser melting, laser beam melting, low-alloyed steel, Bainidur AM, material properties, phase formation, tempering stability, bainitic steels, additive manufacturing

## Abstract

Laser-based powder bed fusion of metals (PBF-LB/M) is an emerging technology with enormous potential for the fabrication of highly complex products due to the layer-wise fabrication process. Low-alloyed steels have recently gained interest due to their wide potential range of applications. However, the correlation between the processing strategy and the material properties remains mostly unclear. The process-inherent high cooling rates support the assumption that a very fine martensitic microstructure is formed. Therefore, the microstructure formation was studied by means of scanning electron microscopy, hardness measurements, and an analysis of the tempering stability. It could be shown that additively manufactured Bainidur AM samples possess a bainitic microstructure despite the high process-specific cooling rates in PBF-LB/M. This bainitic microstructure is characterized by an excellent tempering stability up to temperatures as high as 600 °C. In contrast to this, additively manufactured and martensitic-hardened specimens are characterized by a higher initial hardness but a significantly reduced tempering stability. This shows the potential of manufacturing products from Bainidur AM for high-temperature applications without the necessity of a post-process heat treatment for achieving the desired bainitic microstructure.

## 1. Introduction

Bearing and gear applications have an extremely high demand regarding the underlying material properties as a high-impact toughness is required while also providing a sufficient material hardness and wear resistance [1]. The material properties of parts are highly dependent on the underlying microstructure, especially the phase distribution [2]. In steels, for example, a high ductility is achieved through the austenitic or pearlitic phase, while the martensitic phase favors an elevated hardness at the cost of ductility [3]. A trade-off between these two phases is possessed by bainite, which is characterized by a material hardness comparable to tempered martensite [4]. The ductility of bainite is typically higher compared to that of martensite. When generating the bainite, however, a complex heat treatment with adjusted cooling conditions and prolonged holding times is typically necessary [5]. The heat treatment parameters are again dependent on the chemical composition of the material system [6]. Additive manufacturing technologies such as laser-based powder bed fusion (PBF-LB/M) and directed energy deposition (DED-LB/M) are known for their high cooling rates, which can exceed 10^3^ K/s for both processes [7,8]. This would typically result in the formation of a martensitic phase in carbon steels [9]. In recent years, the Deutsche Edelstahlwerke Specialty Steels GmbH (Krefeld, Germany) developed a low-alloyed case-hardening steel named Bainidur AM. The chemical composition of this alloying system was adjusted to favor the formation of a bainitic microstructure in the PBF-LB/M process [10]. Kelliger et al. [11] have used this material to manufacture nozzles with optimized cooling channels. However, no studies on the microstructural properties were performed within this work. Apart from that, Bartels et al. [12] showed the general processability of Bainidur AM by means of DED-LB/M. These early investigations indicate that at least a partial bainitic microstructure is formed during the DED-LB/M. However, extensive research on this material system and the corresponding phase formation in this material system is not available in the literature up to now.

Due to their low carbon content, case-hardening steels typically possess a good weldability, making them suitable for welding-based processes [13]. First studies on the processing of this group of steels by means of PBF-LB/M have already been performed. Independent researchers, e.g., Kamps [14], Schmitt et al. [15], and Bartels et al. [16], show that defect-free parts from 16MnCr5 can be manufactured additively. Schmitt et al. [17] also state in another work that a predominant bainitic microstructure with shares of martensite is formed when processing the low-alloyed steel 16MnCr5 by means of PBF-LB/M. However, thorough investigations on the microstructure are missing within this work as the focus was laid on studying the process chain for manufacturing gears using the PBF-LB/M process. Aumayr et al. [18] investigated the processing of the low-alloyed steel Böhler E185 AMPO, also using the PBF-LB/M process. The carbon content of this material is around 0.2 wt.%. Here, a defect-free processing was possible for different parameter combinations. This material was also characterized by an excellent impact toughness of around 140 J. Tensile strength and elongation at break were determined to be around 1150 N/mm^2^ and 15%, respectively. This was attributed to the bainitic-martensitic microstructure formed during the PBF-LB/M process. After hardening, a higher tensile strength at the cost of a reduced ductility could be observed, which further underlines these findings that martensite is not the main phase within the microstructure. Zumhofen et al. [19] investigated the PBF-LB/M of 30CrNiMo8. This material is characterized by a higher carbon content compared to 16MnCr5, which could potentially favor defect formation during build-up. Their experiments show that defect-free specimen with a high relative part density can be fabricated using a platform preheating of 300 °C. The as-built material is characterized by an acicular microstructure compared to the same material in the quenched and tempered state. Damon et al. [20] studied the impact of the process intrinsic heat treatment on the mechanical properties of the quench and tempering (QT) steel 42CrMo4. Within their work, they found that a similar hardness of around 40 HRC could be observed when tempering the quenched material at 450 °C. Considering the high cooling rates of the PBF-LB/M process and the preheating temperature of around 200 °C, a significantly higher hardness of around 650 HV should be present after fabrication. Instead, the as-built microstructure possesses finely dispersed carbides, which are typical for tempered martensite. The authors assume that a reheating and tempering of lower layers takes place during the manufacturing process, which could explain the tempered martensitic structure. Another work by Beer et al. [21] investigated the processing of the case-hardening steel M50NiL by means of PBF-LB/M and compared the resulting material properties with the ones obtained for conventionally manufactured M50NiL. In both cases, similar microstructural and mechanical properties were obtained. Furthermore, additively manufactured samples mastered fatigue life testing without premature failure.

The literature review shows that parts from low-alloyed steels can be manufactured successfully using the PBF-LB/M process. However, fundamental investigations on the microstructure formation are still missing for this material class. These studies need to be performed to fully understand the phase formation and the resulting material properties after PBF-LB/M to fully exploit the benefits of the process-specific fine microstructure.

The goal of this work is to study the influence of different processing conditions and strategies on the phase formation in the low-alloyed steel Bainidur AM (1.7980; 18MnCrMoV4-8-7) in PBF-LB/M. As the name suggests, the material was designed to form bainite during additive manufacturing processes. It is assumed that a predominantly bainitic microstructure can be formed in PBF-LB/M despite the high cooling rates in the order of 10^3^ to 10^5^ K/s [7,8] of this process. Furthermore, the tempering stability will be analyzed as an indicator for a bainitic microstructure.

## 2. Materials and Methods

The powder material Bainidur AM (Deutsche Edelstahlwerke, Germany) was used for the underlying experiments. All experiments were performed on an AconityMINI (Aconity 3D, Germany) equipped with a 1 kW fiber laser. The average particle size distribution of the powder batch ranged from 15 to 45 µm (d_10%_ = 18.28 µm, d_50%_ = 32.72 µm, d_90%_ = 46.58 µm, measured using a Camsizer). Table 1 lists the chemical composition of the material system according to the supplier’s certificate.

The powder material is characterized by a low carbon content of 0.22 wt.%, which indicates that the material should be processed successfully without cracks using PBF-LB/M. An ELEMENTRAC CS-i (ELTRA GmbH, Haan, Germany) analyzer was used to validate the carbon content of the powder material. Furthermore, optical emission spectroscopy (OES) was used for determining the chemical composition of the material in the as-built state. A SPECTROMAXx (SPECTRO Analytical Instruments GmbH, Kleve, Germany) was used for these measurements. The composition was determined in the center of the specimen.

**Figure 1:**
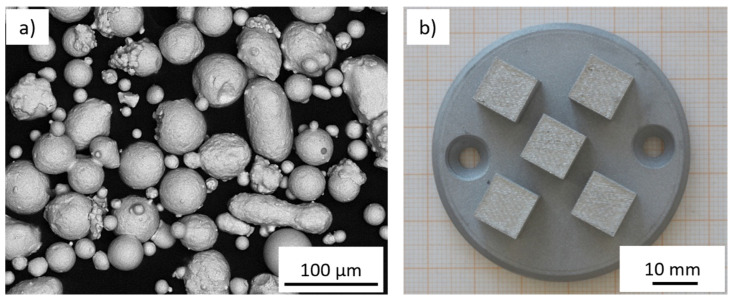
Morphology of the powder materials used (**a**) with a nominal size from 15 to 45 µm and (**b**) an exemplary design of the build job on the AconityMINI.

Figure 1a presents SEM images of the powder material used within this work, while Figure 1b shows an exemplary build job consisting of five cubic specimens.

Primarily spherical particles can be observed with shares of sporadic, irregularly shaped particles with a longish form. A good flowability can be assumed, which is necessary for the supply of the powder layer during the PBF-LB/M process.

### 2.1. Identification of Process Window

For identifying the process window, the three dominant parameters of laser power, scanning speed, and hatch distance were varied in a wide range. Within these experiments, the contour parameters were maintained constant. The process parameter range was deduced using cubic specimens with dimensions of 10 × 10 × 10 mm. Laser spot size and layer thickness were maintained constant throughout all experiments. The investigated range of parameters is listed in Table 2.

The layer thickness was maintained constant at 60 µm per layer. The default scanning direction was turned by 67° after every layer on the AconityMINI, a commonly used scanning strategy in PBF-LB/M. All samples were manufactured without platform preheating.

### 2.2. Sample Preparation and Analysis

**Figure 2:**
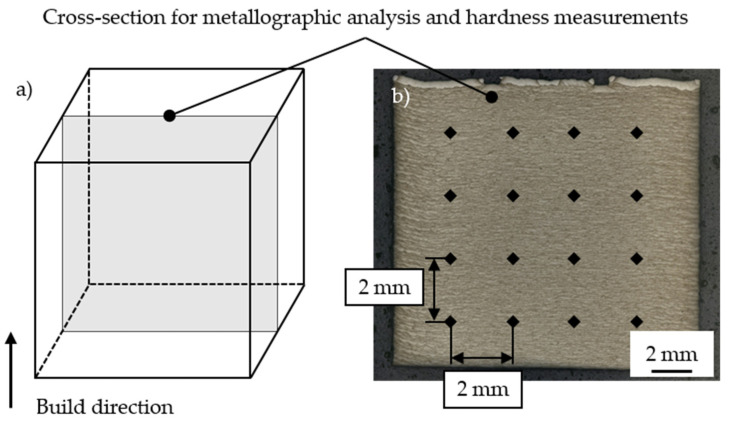
Experimental approach for (**a**) sample preparation for analysis of the relative part density and hardness measurements and (**b**) measurement pattern for determining the material hardness.

All specimens were embedded, ground, and polished to analyze the relative part density and material hardness on cross-sections. The approach for the sample preparation is presented in Figure 2.

The relative part density was determined on cross-sections in x-z-direction within the center of the specimen. For this, the specimens were ground down to approximately 5 mm until the center was reached. The relative part density was determined based on the binarization method. An automatic threshold was set to distinguish pores or other defects (dark) from the solidified material (bright). The corresponding pixel ratio was chosen as relative part density. Due to the high relative part density at low magnifications, the relative part density was also determined in the most porous-appearing region for larger magnifications.

The Vickers hardness (HV1) was determined in the central region of the specimens for the preliminary investigations using a Qness indentation tester (ATM Qness GmbH, Mammelzen, Germany). Therefore, a 4 × 4 pattern was placed in the center of the specimens. The distance between two measurement points was set to 2 mm. The average material hardness was calculated from the resulting 16 measuring points.

Microstructure analysis was performed by means of optical light microscopy using a Leica DM4 M. Therefore, the samples were etched for three seconds using a <5% Nital solution to reveal the microstructure.

A Tescan Vega and a Mira3 SEM were utilized for scanning electron microscopy (SEM) to generate images of the cross-sections with larger magnifications of up to 35,000 times. X-ray diffraction (XRD) was performed according to [22] using a D8 Discover (Bruker Corporation, Billerica, MA, USA) system equipped with a Lynxeye 1D-detector.

### 2.3. Post-Process Heat Treatment Strategy

Different post-process heat treatment strategies were examined to assess the formation of bainite. In the first steps, one reference series with a medium-volume energy density (VED, $VED=\frac{P}{v*h*t}$ in J/mm^3^) was exposed to both a tempering and a quenching and tempering heat treatment. One half (approximately 5 × 10 × 10 mm) of the sample was annealed in the as-built state, while the other half (approximately 5 × 10 × 10 mm) was tempered from the quenched state. Table 3 presents the corresponding parameters for heat treatment.

Austenitization was performed at 920 °C for 30 min, as stated in the supplier’s data sheet for the conventional bainitic steel Bainidur 7980 CN [23]. An oil bath was used for quenching the specimens. The corresponding tempering temperatures varied between 150 and 600 °C as a high-temperature stability was expected for the as-built Bainidur AM samples. All samples were kept in the oven for 1 h. After heat treatment, the samples were air-quenched. The quenching experiments were performed in a furnace of type N 31/H (Nabertherm GmbH, Germany). Tempering was carried out in an N 30/85SHA (Nabertherm GmbH, Germany) oven. All experiments were performed under nitrogen gas atmosphere to avoid oxidation.

## 3. Results

This section is divided into three main parts. First, the results on the process window are presented, in which nearly defect-free specimens with a relative part density above 99.7% can be manufactured. These investigations are followed by a fundamental analysis on the microstructure formation and the material hardness. In the final step, the tempering stability of the additively manufactured material is assessed based on the material hardness and microstructural properties.

### 3.1. Process Window for the Defect-Free Fabrication of Bainidur AM

**Figure 3:**
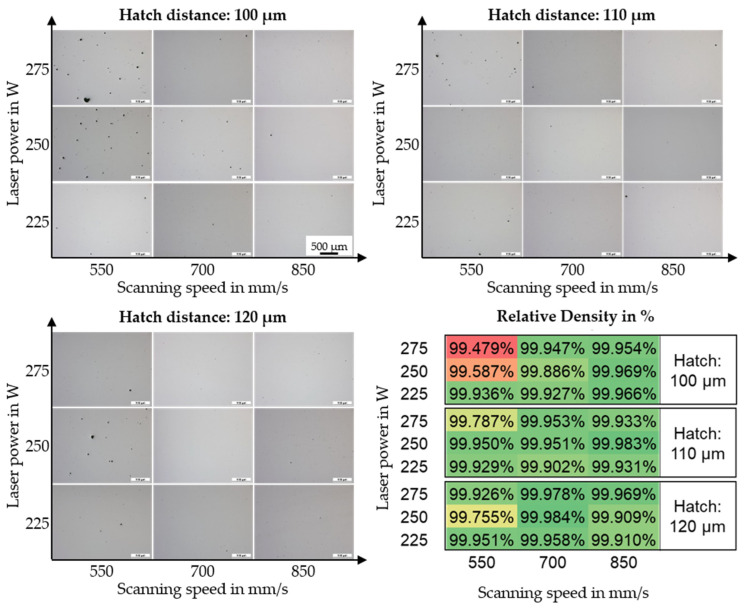
Results of the optical density analysis for the different processing parameters on the AconityMINI machine. The scale is magnified exemplary only once (225 W–850 mm/s–100 µm). Red indicates a lower relative part density, green indicates a higher relative part density.

**Figure A1:**
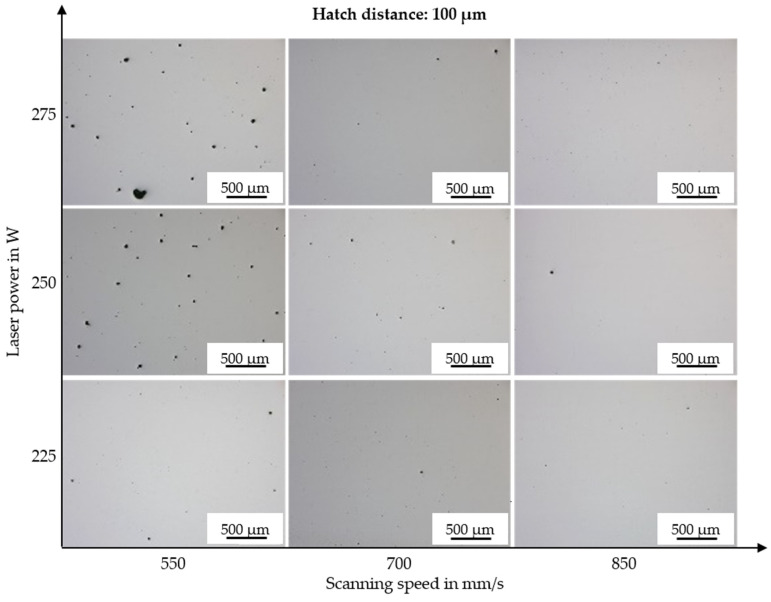
Cross-sections of the specimens manufactured with a hatch distance of 100 µm.

**Figure A2:**
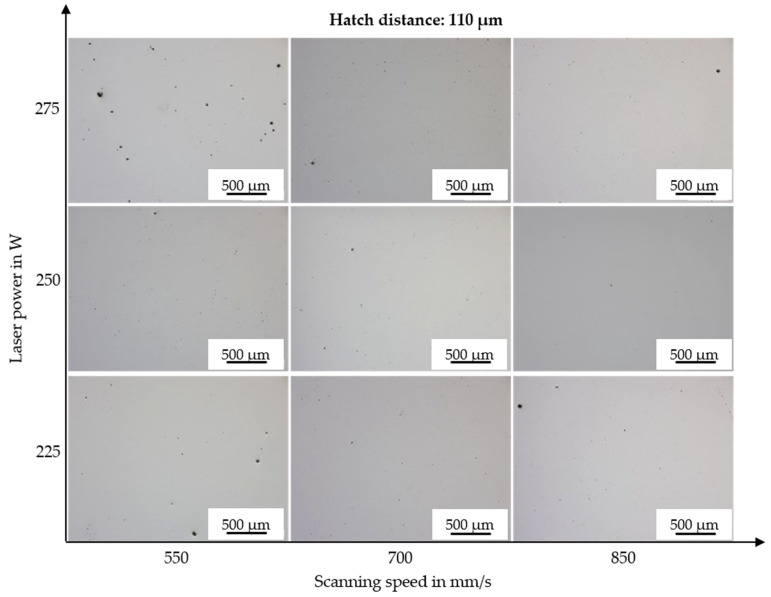
Cross-sections of the specimens manufactured with a hatch distance of 110 µm.

**Figure A3:**
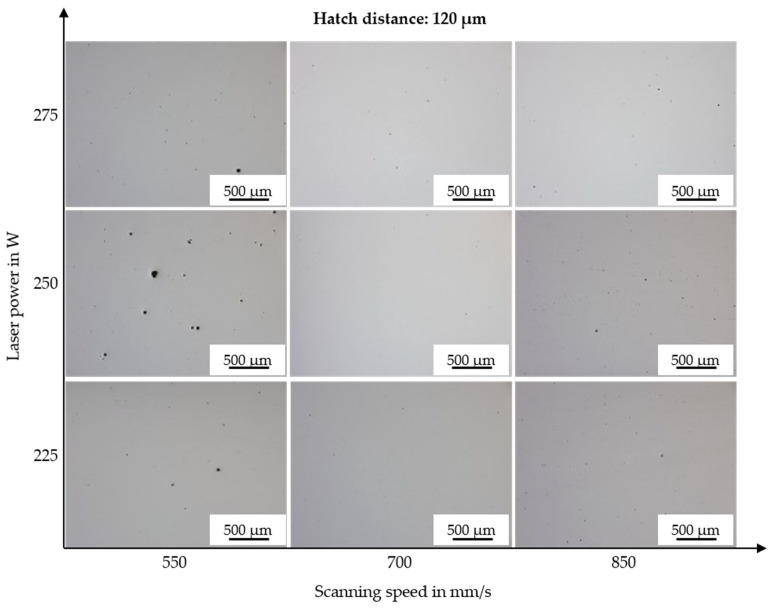
Cross-sections of the specimens manufactured with a hatch distance of 120 µm.

The AconityMINI system was used for identifying the potential process window. Due to the small build envelope, screening investigations can be performed already for low amounts of powder. Three different hatch distances (100, 110, and 120 µm) were studied for three different laser powers (225, 250, and 275 W) and three different scanning speeds (550, 700, and 850 mm/s). In each build job, five samples were manufactured. Figure 3 presents the results on the relative part density for the investigated parameter window. A larger representation of the different cross-sections can be found in Appendix A (Figure A1, Figure A2 and Figure A3).

**Figure 4:**
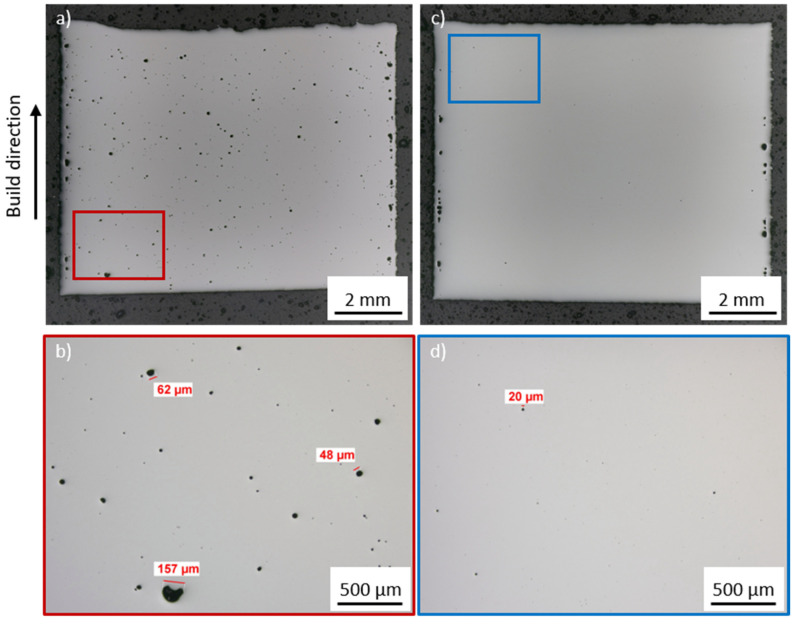
Exemplary (**a**,**c**) cross-sections and (**b**,**d**) magnified regions for determining the relative part density in the worst apparent regions of the specimen for (**a**,**b**) 275 W–550 mm/s–100 µm and (**c**,**d**) 275 W–700 mm/s–110 µm.

The stated relative part density was determined within the areas of the cubic samples that appear to have the highest number of pores or defects (this approach can also be seen in Figure 4). Overall, Bainidur AM is characterized by a good processability by means of PBF-LB/M as part densities above 99.7% can be achieved for most parameter combinations. Figure 4 presents the relative part density for two different parameter combinations. One specimen (Figure 4a) was manufactured using a high VED. The other specimen (Figure 4c) was manufactured using a lower volume energy density. Figure 4b,d show a magnified region of the specimen, which is characterized by a higher number of pores compared to the rest of the specimen.

A high energy density tends to result in an increased defect formation within the specimen. Possible explanations for this are the increased keyhole tendency during the laser welding process [24]. This correlates well with the increased size of these pores, which regularly exceed 50 µm. A lowered energy density results in fewer pores, which, if evident, typically fall below a size of 20 µm. All in all, the porosity is distributed homogeneously within the specimen. The contour region is characterized by a high porosity, independent of the used parameter set. However, this can be attributed to the fact that the contour parameters have not been optimized within this work.

### 3.2. Microstructure and Material Hardness

**Figure 5:**
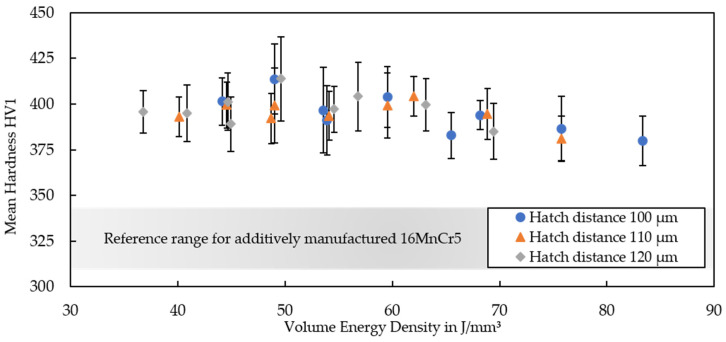
Mean hardness of test cubes manufactured on the AconityMINI systems using the different process parameters. The hardness of additively manufactured 16MnCr5 according to [15] is provided as a reference. Information on the respective standard deviations can be found in Appendix A.

Building on the previous investigations, the material hardness and microstructural properties of the additively manufactured specimens were investigated. Figure 5 shows results on the hardness for the material processed on the AconityMINI machine.

Bainidur AM is characterized by a homogeneous material hardness over a wide range of parameters. The trend indicates that the material hardness decreases for increasing VEDs exceeding 70 J/mm^3^. This appears logical, as higher VEDs—often due to lower scanning speeds or higher laser powers—typically result in a coarser microstructure compared to lower VEDs [25]. Furthermore, these samples possess a higher porosity, which was caused by overheating and keyhole formation during build-up. Apart from the highest VEDs, the material hardness remains constant for a wide parameter window, which can be helpful when manufacturing larger parts in the future to avoid a geometry-specific overheating. The hardness surpasses that of additively manufactured 16MnCr5 by up to 70 HV1 on average [15,16], showing the enormous potential of the low-alloyed steel Bainidur AM for the manufacturing of construction parts. This improved hardness can at least be partially explained by the higher carbon content of Bainidur AM. Apart from that, no significant differences between the specimens could be observed and all specimens were characterized by a continuous and homogeneous hardness in the build direction.

**Figure 6:**
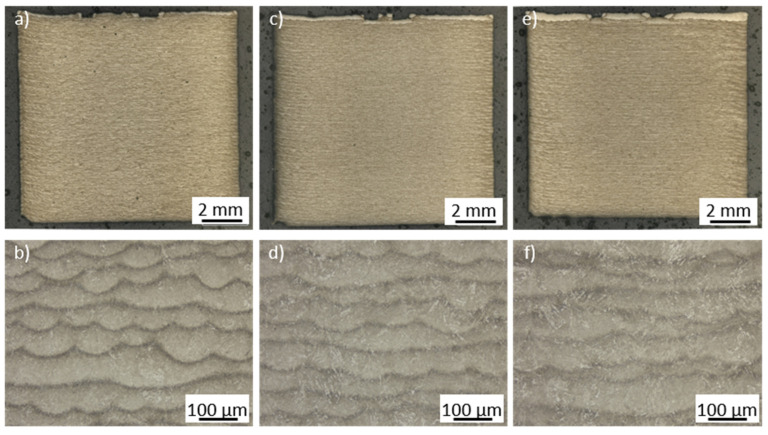
Etched cross-sections for samples manufactured using a (**a**,**b**) low (36.8 J/mm^3^), (**c**,**d**) medium (54.1 J/mm^3^), and (**e**,**f**) high VED (75.8 J/mm^3^).

Next, optical analysis was performed on the etched cross-sections. The obtained results of the cross-sections for three different VEDs are presented in Figure 6. 

**Figure 7:**
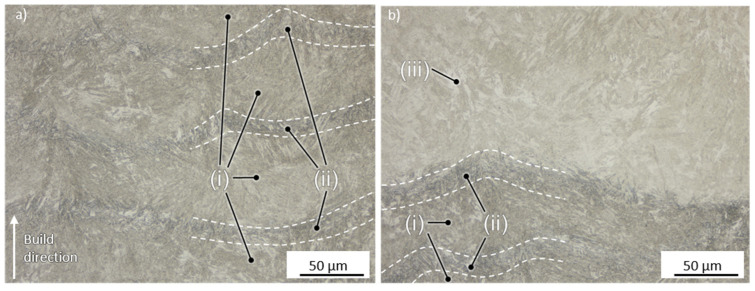
Different microstructural regions of PBF-LB/M specimens (**a**) in the core with the fusion zone (i) and heat-affected zone (ii) and (**b**) in case (iii), manufactured using a medium VED of 54.1 J/mm^3^.

Similar microstructural features were observed at all three VEDs. For low VEDs, the welding depth is reduced compared to the higher VEDs. The bright region of the top layers also increases for higher energy inputs, which underlines the correlation between energy input and weld depth. Apart from that, the lack of visible differences was also supported by the average hardness values, which only increased slightly for higher cooling rates. In all cases, however, different regions within the specimen can be observed. XRD measurements show no correlation between the applied VED and the retained austenite (RA) content, as the RA content remained constant at around 7% to 9%. This was studied for three different VEDs. Figure 7 shows two exemplary cross-sections in the center and in the top section of a specimen manufactured using a medium VED of 54.1 J/mm^3^.

**Figure 8:**
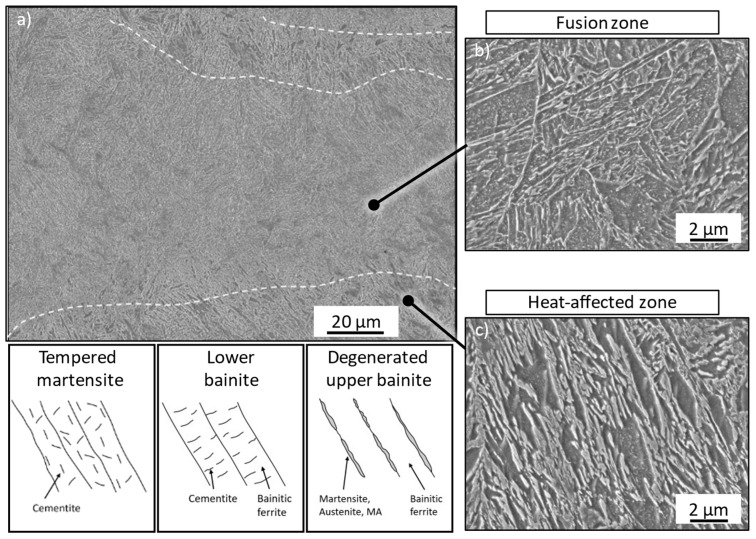
SEM images of the (**a**) fusion zone and the heat-affected zone within the core of the specimen as well as corresponding magnification of the (**b**) fusion zone and (**c**) the heat-affected zone. Exemplary illustrations of the apparent microstructure according to [26].

Three different regions can be identified within the specimen. The top layer (iii) appears brighter under the microscope as the heat input is lower due to the absence of the continuous reheating during the layer-wise manufacturing process. In contrast to this, the core region can be divided into the fusion zone (FZ, i) and the heat-affected zone (HAZ, ii), with the FZ being brighter than the HAZ. The FZ also appears to possess a finer grain than the surrounding HAZ. Correspondingly, SEM analysis was performed in these regions. Figure 8 presents the results for the FZ and HAZ. 

As expected, a different microstructure was formed within the HAZ and the FZ. The fusion zone is characterized by a more lath-like structure with carbides finely dispersed within the laths, which is similar to lower bainite or tempered martensite. Due to this fine carbide dispersion, a clear distinction between martensite (random orientation) and bainite (targeted orientation) is barely possible. In contrast, the heat-affected zone appears coarser, with areas appearing such as bainitic ferrite with coarse, partially segmented films between these laths. This seems like a degenerated upper bainitic structure. The less pronounced etching response and smooth surfaces of these films also suggest the presence of austenite or martensite in these areas. This region is further characterized by predominant carbide precipitations at the lath boundaries and less promoted carbide dispersion within the grain. The high process-specific cooling rates and the corresponding fine microstructure make it hard to assess what type of microstructure is underlying. Therefore, an additional analysis on the tempering stability was performed.

### 3.3. Tempering Stability as an Indicator for a Bainitic Microstructure

In theory, the bainite possesses a higher tempering stability than martensite [27]. To better assess the microstructure in the as-built state, a tempering series was started. Prior to this series, the chemical composition of the as-built specimens was determined by optical emission spectroscopy in the center of the specimen. The results are shown in Table 4.

**Figure 9:**
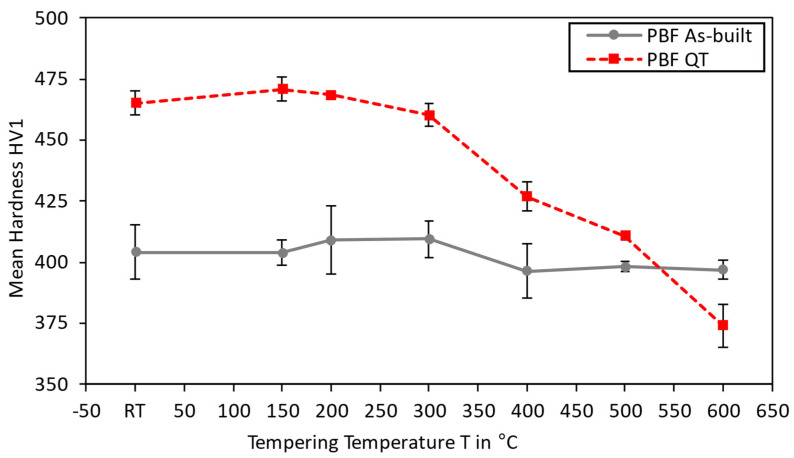
Material hardness for different tempering temperatures in the as-built as well as quenched and tempered states. The specimens were manufactured using a medium VED (54.1 J/mm^3^).

Comparing the chemical composition of the sample with the powder, a slight decrease in carbon content from 0.22 wt.% (powder) to 0.20 wt.% (sample) could be observed. This decarburization can be attributed to the high temperatures during the manufacturing process, which was also reported for the martensitic steel AF9628 by Seede et al. [28]. Figure 9 presents the results on the tempering studies for as-built and tempered (AT) as well as quenched and tempered (QT) specimens.

**Figure 10:**
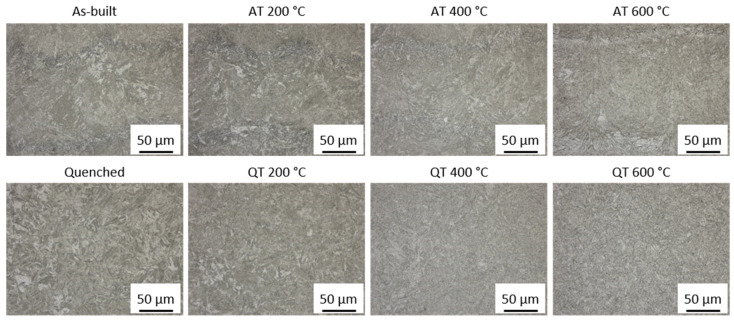
Etched cross-section of (**top**) as-built and tempered as well as (**bottom**) quenched and tempered specimens. The specimens were manufactured using a medium VED (54.1 J/mm^3^).

The QT specimens possess the highest material hardness (470 HV1) at room temperature. Increasing the tempering temperature results in a hardness decrease, a common effect observed for tempered martensite in low-alloyed steels [29]. The corresponding material hardness falls as low as 370 HV for the highest tempering temperature of 600 °C. In contrast, as-built and tempered specimens possess a homogeneous material hardness of 405 HV1 at all temperatures. This indicates that a primarily bainitic microstructure is present after manufacturing. Santajuana et al. [30] and Peet et al. [31] have both found a similar high-temperature stability of bainite up to tempering temperatures of approximately 500 °C for steels with a higher carbon content. The work by Kafadar et al. [32] also indicates that the alloying elements, especially molybdenum, significantly affect the tempering stability. Sourmail et al. [33] report similar findings on the effect of vanadium. As both these alloying elements are present in the material Bainidur AM, it can be expected that these alloys result in carbide formation during cooling. Figure 10 presents images of the corresponding etched cross-sections after the respective tempering heat treatment.

**Figure 11:**
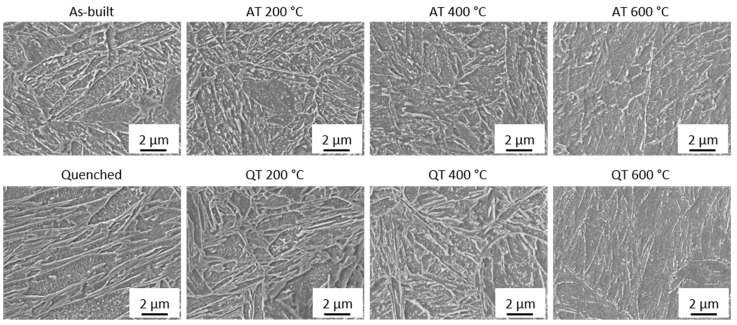
SEM images of the center region of the specimens in (**top**) as-built and tempered as well as (**bottom**) quenched and tempered states. The specimens were manufactured using a medium VED (54.1 J/mm^3^).

**Figure A4:**
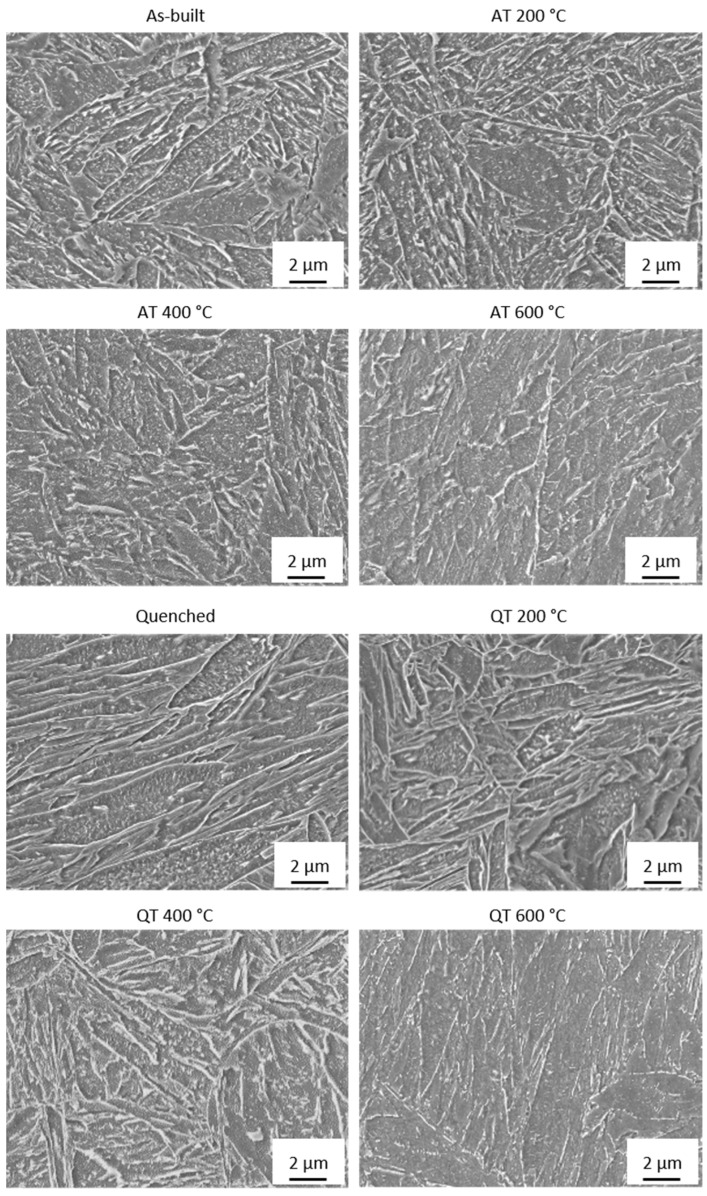
SEM images (from Figure 11) of the as-built and tempered as well as quenched and tempered specimens.

In the as-built state, a clear distinction between the fusion zone and the heat-affected zone is present, even at tempering temperatures of 600 °C. Furthermore, a very fine microstructure can be observed throughout all temperatures. Increasing the tempering temperature results in a decomposition of the retained austenite (white clusters in Figure 10, as-built and AT 200 °C) within the specimen. For the QT specimens, an obvious change in microstructure is evident, which could explain the hardness drop. While the as-built specimens still possess the PBF-specific structure as the contour of the weld tracks can still be observed up to tempering temperatures of 600 °C, this structure can no longer be determined for the quenched specimens. Figure 11 shows additional SEM images of the microstructure of these specimens. A larger representation of these figures is shown in Appendix A (see Figure A4).

The as-built and tempered samples possess finely dispersed carbides within the grains at all temperatures. These carbides can be the reason for the good thermal stability of the as-built specimens. At a tempering temperature of 600 °C, however, these carbide structures are harder to identify and barely present. Mohr et al. [34] have found the global temperatures at the surface of the specimen to fall below 250 °C for 10 mm specimens within their work (see Figure 4 in [34]). Thus, it is unlikely that the underlying microstructure is a tempered martensitic one, as a hardness drop-off would be expected when exceeding the process-specific temperature (in this case approximately 250 °C). Fine carbides can also be identified in the hardened (and tempered) specimens. However, these specimens were characterized by a hardness drop-off, which is typically observed when tempering martensite. It is therefore more likely that a bainitic microstructure is underlying in the as-built state, despite the optical similarities in the as-built and quenched states.

XRD analyses show that the retained austenite dropped from around 7.5% to around 1.1% after tempering the as-built specimens for 1 h at 600 °C. This falls below the detection limit of the XRD, which is around 2%, indicating that the retained austenite is almost fully transformed into bainite and possibly secondary carbides.

Overall, an extremely favorable, bainite-like microstructure is present after the PBF-LB/M process. Even though shares of retained austenite and even martensite cannot be ruled out, a consistent hardness even after being exposed to the highest tempering temperatures is observed. This indicates that a microstructure with bainite-like properties can be obtained after the PBF-LB/M process.

## 4. Conclusions

The low-alloyed steel Bainidur AM can be successfully processed using a wide range of parameters by means of PBF-LB/M. It was found that a predominantly bainitic or at least bainite-like microstructure could be generated in the as-built state despite the PBF-specific high cooling rates. This was validated by analyzing the material hardness, the tempering stability as an indicator for a bainitic microstructure, SEM analysis of the microstructure, and XRD measurements of the retained austenite content. The main findings of this work are:

- In the absence of elevated preheating temperatures, a fine bainitic microstructure was formed during PBF-LB/M.
- A structure similar to lower bainite was formed in the fusion zone, while the heat-affected zone appeared more like an upper bainitic structure.
- The as-built samples were characterized by a material hardness of around 400 HV1, surpassing that of other additively manufactured, low-alloyed steels.
- Samples manufactured from Bainidur AM possessed an excellent tempering stability, characterized by a homogeneous hardness up to tempering temperatures as high as 600 °C.
- Applying a post-process heat treatment helped in reducing the minor retained austenite content through the transformation into bainitic structures.

Overall, the material properties are very promising as Bainidur AM possesses an excellent hardness for a low-alloyed, case-hardening steel. Further in-depth analysis on the mechanical properties such as tensile strength and impact toughness will be within the scope of future studies to further validate this material system for PBF-LB/M.

## Figures and Tables

**Table 1:** Chemical composition of the Bainidur AM powder batch used within this work.

Batch	Element Content in wt.%								
	C	Si	Mn	P	S	Cr	Mo	Ni	V
Powder	0.22	0.7	1.2	<0.02	<0.02	1.0	0.9	<0.3	<0.15

**Table 2:** Investigated process parameter range.

Process Parameter	Parameter Range
Laser Power, P (W)	225–250–275
Scanning Speed, v (mm/s)	550–700–850
Hatch Distance, h (µm)	100–110–120
Layer Thickness, t (µm)	60
Laser Spot Diameter (µm)	105
Shielding Gas	Argon

**Table 3:** Heat treatment parameters.

Tempering Temperature	As-Built and Tempered	Quenched and Tempered
150 °C	AT150	QT150
200 °C	AT200	QT200
300 °C	AT300	QT300
400 °C	AT400	QT400
500 °C	AT500	QT500
600 °C	AT600	QT600

**Table 4:** Chemical composition of the powder material according to the supplier’s certificate and after PBF-LB/M determined by OES.

Batch	Element Content in wt.%						
	C	Si	Mn	Cr	Mo	Ni	V
Powder	0.22	0.7	1.2	1.0	0.9	<0.3	<0.15
As-built	0.20	0.67	1.35	1.01	0.86	0.09	0.02

## Data Availability

Raw data and material are available upon request.
